# Experimental confirmation of long hyperbolic polariton lifetimes in monoisotopic (^10^B) hexagonal boron nitride at room temperature

**DOI:** 10.1063/5.0061941

**Published:** 2021-09

**Authors:** Georges Pavlidis, Jeffrey J. Schwartz, Joseph Matson, Thomas Folland, Song Liu, James H. Edgar, Josh D. Caldwell, Andrea Centrone

**Affiliations:** 1 Nanoscale Spectroscopy Group, Physical Measurement Laboratory, NIST, Gaithersburg, Maryland 20899, USA; 2 Institute for Research in Electronics and Applied Physics, University of Maryland, College Park, Maryland 20742, USA; 3 Mechanical Engineering, Vanderbilt University, Nashville, Tennessee 37235, USA; 4 Tim Taylor Chemical Engineering, Kansas State University, Manhattan, Kansas 66506, USA

## Abstract

Hyperbolic phonon polaritons (HPhPs) enable strong confinements, low losses, and intrinsic beam steering capabilities determined by the refractive index anisotropy—providing opportunities from hyperlensing to flat optics and other applications. Here, two scanning-probe techniques, photothermal induced resonance (PTIR) and scattering-type scanning near-field optical microscopy (s-SNOM), are used to map infrared (6.4−7.4μm) HPhPs in large (up to $\left.120\times 250\;\mu m^{2}\right)$ near-monoisotopic >99%B10) hexagonal boron nitride (hBN) flakes. Wide (≈40μm) PTIR and s-SNOM scans on such large flakes avoid interference from polaritons launched from different asperities (edges, folds, surface defects, etc.) and together with Fourier analyses $\left(0.05\;\mu m^{-1}\right.$ resolution) enable precise measurements of HPhP lifetimes (up to $\left.\approx 4.2\;ps\right)$ and propagation lengths (up to ≈25 and ≈17μm for the first- and second-order branches, respectively). With respect to naturally abundant hBN, we report an eightfold improved, record-high (for hBN) propagating figure of merit (i.e., with both high confinement and long lifetime) in ≈99%B10 hBN, achieving, finally, theoretically predicted values. We show that wide near-field scans critically enable accurate estimates of the polaritons’ lifetimes and propagation lengths and that the incidence angle of light, with respect to both the sample plane and the flake edge, needs to be considered to extract correctly the dispersion relation from the near-field polaritons maps. Overall, the measurements and data analyses employed here elucidate details pertaining to polaritons’ propagation in isotopically enriched hBN and pave the way for developing high-performance HPhP-based devices.

## INTRODUCTION

Guiding and confining light to sub-wavelength dimensions is a key focus of nanophotonics since enhanced light-matter interactions at the nanoscale enable new spectroscopic,^1–4^ nonlinear,^5–8^ and other advanced applications, as well as serve as the basis for miniaturizing existing optical devices.^9–11^ One strategy to exert such nanoscale control is to harness hybrid excitations between light and coherent charge oscillations in materials, known as polaritons. While plasmon polaritons can be engineered across a broad spectral range, the short charge carrier scattering times in metals (≈fs) result in high losses that limit their applicability, even in the mid-IR.^12,13^ The discovery of hyperbolic phonon polaritons (HPhPs) in naturally occurring materials, such as quartz,^14^ hexagonal boron nitride^15,16^ (hBN), and MoO_3_,^17–19^ has generated significant interest due to their remarkable optical properties and long lifetimes (up to ≈12 ps in MoO_3_,^19^ Rather than being bound to the interface between the polaritonic medium and surrounding dielectric, HPhPs propagate through the volume of highly anisotropic materials characterized by permittivity values that are opposite in sign along principal axes. For extremely anisotropic polar materials, this condition is satisfied in regions between transverse optic (TO) and longitudinal optic (LO) phonon pairs, known as the Reststrahlen bands.^12,20^

The losses in HPhPs are determined by phonon scattering rather than the much faster scattering rates of free-carriers, providing lower losses than plasmon polaritons, which is intrinsically advantageous for realizing waveguides and hyperlenses^21–24^ among other devices. Isotopic enrichment is a useful strategy to increase phonon lifetimes in materials composed of atoms with multiple naturally occurring isotopes (such as hBN) since, in addition to point defects (i.e., impurities), the scattering of optic phonons depends strongly on isotopic disorder.^24,25^ For example, intrinsic HPhP lifetimes (τ), propagation lengths $\left(L_{p}\right)$, and figures of merit (i.e., the polaritons quality factor, Q in Fourier space) were predicted to increase for hBN^24–26^ containing isotopically pure boron 100%B10, to range between τ≈3.3^24^ and 7.5 ps^26^ compared to naturally abundant boron (80%B11, 20%B10 , τ≈1.5ps)^24^ at room temperature. Increased lifetimes and propagation lengths have been experimentally confirmed in near-monoisotopic (≈98.7%B10,^26^ ≈99.22%B10,^27^ and ≈99.2%B11^28^) samples using scattering-type near-field optical microscopy (s-SNOM). However, the measured values at room temperature (τ<1.2ps, $L_{p}<4\;\mu \text{m}$, Q≈40 for 98.7%B10;^26^ $L_{p}<8\;\mu \text{m}$, Q≈50 for 99.22%B10;^27^ and $L_{p}<5\;\mu \text{m}$, Q≈30 for 99.2%B11)^28^ were significantly lower than theory predictions.^24^ Giles *et al*.^26^ proposed that for a flake ≈10μm in width, HPhPs launched from opposite edges can interfere in the middle, leading to discrepancies with predicted values. In addition, here, we will show that the limited scan size (comparable to $L_{p}$) afforded by smaller flakes provides an insufficient accuracy for evaluating $L_{p}$ and τ.^26^

In this paper, we leverage photothermal induced resonance (PTIR)^29,30^ measurements using a tapping-mode heterodyne detection scheme^31^ to measure, with nanoscale resolution, HPhPs propagating in isotopically enriched ≈99%B10 hBN flakes with lateral dimensions up to $120\times 250\;\mu m^{2}$. Fourier analyses of real-space PTIR images yield the HPhP dispersion relation with lifetimes up to ≈4.2 ps, propagation lengths up to ≈25μm, and record-breaking Q (up to Q≈90), approaching the values previously predicted by theory.^24–26^ This confirms the predicted dominant role of isotopic disorder, over anharmonic and impurity background decay channels, in determining the lifetimes of optic phonons in hBN.^25^ Not only are those values the highest reported at room temperature for isotopically enriched hBN, but also the propagation lengths and Q reported here exceed the previously reported values $\left(L_{p}\approx 8\;\mu m\right.,Q\approx 60$) for ≈99%B10 at cryogenic temperatures (45 K).^27^ We show that s-SNOM data validate the PTIR measurements and conclude that to properly compare these two nanoscale IR techniques, it is critical to account for the light incident angles with respect to the sample plane and crystal edges that are typically different in the two setups. In addition to HPhPs, we also observe weakly confined modes both within and below the hBN Reststrahlen band that we attribute to light guiding within the hBN flakes.

## EXPERIMENTAL METHODS

Until recently, most of the optical dispersion relations in hyperbolic materials have been determined experimentally using real-space s-SNOM imaging coupled with reciprocal-space data analysis^15,17,21,22,32,33^ or through the use of nanostructures of different sizes by far-field FTIR.^16,34^ Similar to s-SNOM, PTIR^29,30^ is a scanning-probe-based technique that translates the benefits of infrared (IR) spectroscopy to the nanoscale. Rather than measuring the near-field scattered light from an atomic force microscope (AFM) tip in proximity of the sample, as in s-SNOM, PTIR yields nanoscale IR absorption spectra and maps by transducing the light-induced thermal expansion of the sample with the AFM probe tip.^35^ Typically, in PTIR experiments, the probe maintains contact with the sample surface (contact-mode).^36–38^ In this study, however, we leverage the recently introduced PTIR tapping-mode heterodyne measurement paradigm.^31,39,40^ In practice, a piezoactuator drives the cantilever oscillation in resonance with its second bending mode $\left(f_{2}\approx 1550\;kHz\right)$ and in the repulsive regime; the PTIR signal is demodulated at its first bending mode $\left(f_{1}\approx 250\;kHz\right)$ while pulsing the IR laser at $f_{\text{Laser}}=f_{2}-f_{1}\approx 1300\;kHz$. Such a heterodyne scheme is made possible by the nonlinear tip–sample interactions that enable mixing the tapping motion of the cantilever with the sample photothermal expansion.^41^ Importantly, the fast PTIR mechanical signal transduction provides a spatial resolution (≈10nm in tapping-mode^39^ and up to ≈20nm in contact-mode^38,42^) that is largely unaffected by the (slower) heat diffusion processes. This mechanism results in a much higher spatial resolution than can be obtained with temperature-sensitive AFM probes.^43,44^ The PTIR signal is directly proportional to the product of the local sample absorption coefficient^45–47^ and the local electric field,^3,48,59^ enabling identification of chemical groups and materials^31,50–53^ and providing nanoscale near-field maps.^48,49,54,55^ In the context of two-dimensional (2D) materials, PTIR has been used to determine the distribution of chemical groups^52^ and molecular adsorbates^56^ in graphene oxide, to identify the composition of polymeric contaminants trapped in 2D heterostructures,^50^ and to characterize HPhPs in hBN nanostructures^34,57,58^ and MoO_3_^19^ crystals. Specifically, the detection of HPhPs in hBN frusta via PTIR has provided a more complete set of theoretically predicted modes, revealing (dark) excitations^34,57^ undetected with s-SNOM or in far-field measurements. Additional HPhP studies using PTIR could thus be beneficial to understand and engineer hyperbolic media as well as to explore the potential role of hyperbolic polaritons in thermal dissipation at fast timescales.^59^ Although PTIR is generally a sub-diffraction technique, here, it could also be considered as a near-field technique since the tip plays an active role in generating the polariton contrast mechanism.

## RESULTS AND DISCUSSION

**FIG. 1.**
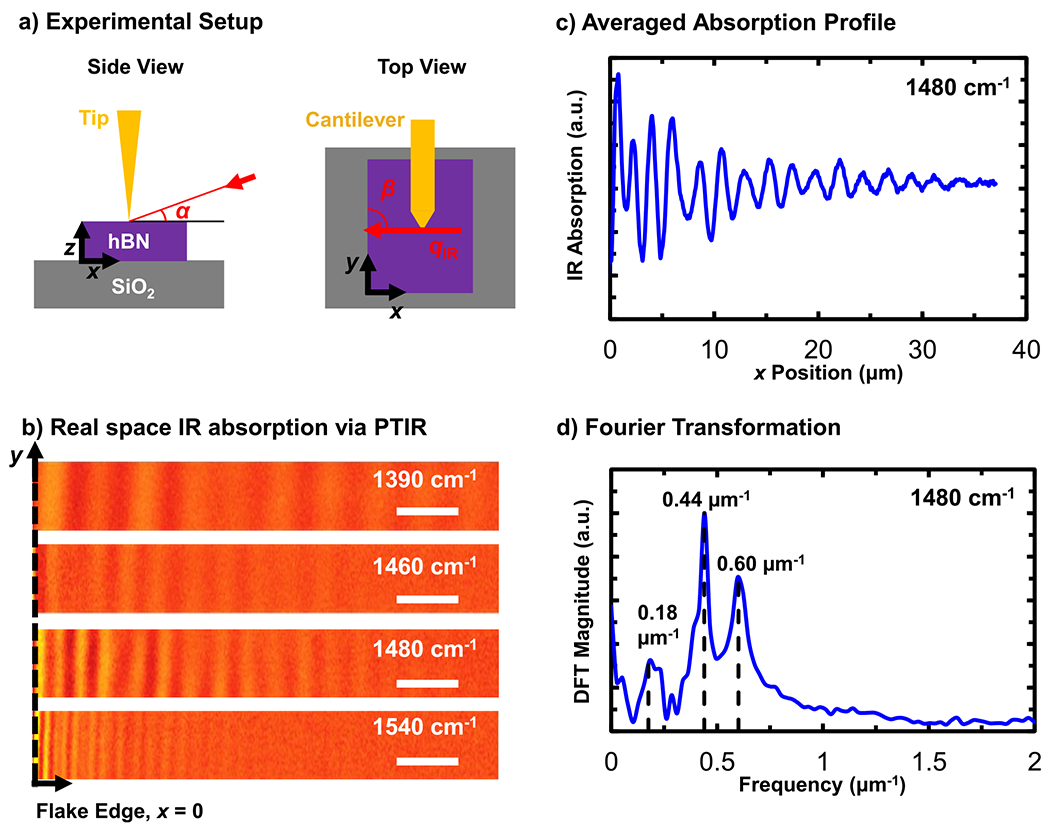
(a) Schematic of the sample illumination in photothermal induced resonance (PTIR) experiments. Infrared light (red arrow with wavevector $q_{\mathrm{IR}}$) is incident upon the sample at an angle of α with respect to the plane of the sample and an angle β with respect to the flake edge. (b) Real-space PTIR images of hyperbolic phonon polaritons (HPhPs) in an isotopically enriched B10≈99% hBN flake (≈147nm thick) obtained by illuminating the sample with different wavelengths. The scale bars represent 5μm. (c) Representative column-wise averaged absorption line profile (measured at $1480\;{cm}^{-1}$) as a function of the flake edge (x=0) distance. (d) Discrete Fourier transform (DFT) of the absorption line profile in (c) revealing multiple HPhP excitation modes at distinct spatial frequencies.

When a hyperbolic material is nanostructured or confined in one or more directions (e.g., in a thin flake), HPhP propagation is permitted only for specific (quantized) momenta that are dictated by the permittivity tensor of the material and its finite size.^57,60^ Typically, light-scattering sites, such as flake edges or the probe tip, are necessary to launch the polaritons (hereafter referred to as edge- or tip-launched, depending on their origin) as these material discontinuities bridge the momentum gap between photons in free-space and the strongly confined, high-momentum polaritons.^61^ While flake edges and the tip scatter light with a continuum of momenta, HPhPs can propagate in the hBN with only a subset of discrete momenta determined by the permittivity tensor. For example, the PTIR images in Fig. 1(b) show the HPhPs propagating for few tens of micrometers within the hBN flake, which are evident as a series of fringes. Edge-launched polaritons propagate and decay in amplitude toward the flake interior, while tip-launched polaritons originate at the probe tip and are detected upon reflection from a flake edge back to the tip. Edge-launched and tip-launched polaritons are a manifestation of the same physical phenomenon; however, tip-launched polaritons (of frequency ω) are detected only after reflecting back to the tip from a material discontinuity (e.g., a flake edge), forming a standing wave interference pattern of frequency 2ω. Therefore, tip-launched polaritons are characterized by fringe periods approximately half that of the corresponding edge scattered polaritons in the PTIR and s-SNOM maps.^62^ This apparent discrepancy is instrumental in nature due to the tip-scanning measurement. Fourier analysis of the real-space PTIR maps [see Fig. 1(c)] more clearly reveals how many HPhP excitation modes contribute to the real-space image as peaks evident in the reciprocal-space power spectra.

In the PTIR experiments, schematically illustrated in Fig. 1(a), the sample was illuminated at an angle, α, with respect to the sample plane $\left(\alpha =20^{\circ }\pm 2^{\circ }\right)$ with light incident from the right side $\left(\approx 90^{\circ }\right.$ with respect to the longitudinal axis of the cantilever). The uncertainty in the incident angle is an estimate of the angle assessment precision. The samples were mounted on a manual rotation stage that enables controlling the angle between the flake edge and the in-plane propagation direction of the incident light, defined as β [see Fig. 1(a)]. The angles α and β are important to consider when comparing measurements obtained under different conditions or different modalities (i.e., PTIR and s-SNOM) as α affects the near-field tip-enhancement strength^63^ and both angles can affect the period of the polariton fringes.^28,64,65^

As reported elsewhere,^66^ the hBN crystal flakes >99%B10 were produced by precipitation from a molten metal solution using isotopically enriched elemental boron (nominally 99.22%B10) provided by a commercial source and nitrogen gas. The samples were prepared by mechanical exfoliation. Crystals of ≈99%B10 isotopically enriched hBN were peeled apart several times using low residue polyimide tape—the tape was then applied onto silicon wafers with a thick (≈1μm) layer of oxide on the surface, which had been cleaned for 5min in an $O_{2}$ plasma. Two flakes with thicknesses of 147±2 and 337±3nm (determined from topography line profiles) were initially studied. The uncertainties in the flake thickness represent one standard deviation in the AFM topography measurements at different flake locations.

To experimentally determine the conditions for the highest launching efficiency, we first compared the PTIR maps obtained with different light polarization (s- or p-polarized, Fig. S1) and flake orientations (Fig. S2). In brief, while we can detect HPhPs using s-polarization (Fig. S1), which has been seldom reported in the literature,^28^ the use of p-polarization (parallel to the plane of incidence) and aligning the flake edge perpendicular and opposed to the light incident direction $\left(\beta =90^{\circ }\right.$, see Fig. 1) provide the most efficient HPhP launching condition. Consequently, this experimental configuration was used to collect all PTIR data and analysis reported in the main text. This orientation, with the AFM fast-scan direction [*x*-axis in Fig. 1(a)] perpendicular to flake edge [*y*-axis in Fig. 1(a)], enables column-wise pixel averaging, reducing each image to a one-dimensional dataset with higher signal-to-noise ratios than a single line profile.^19^

**FIG. 2.**
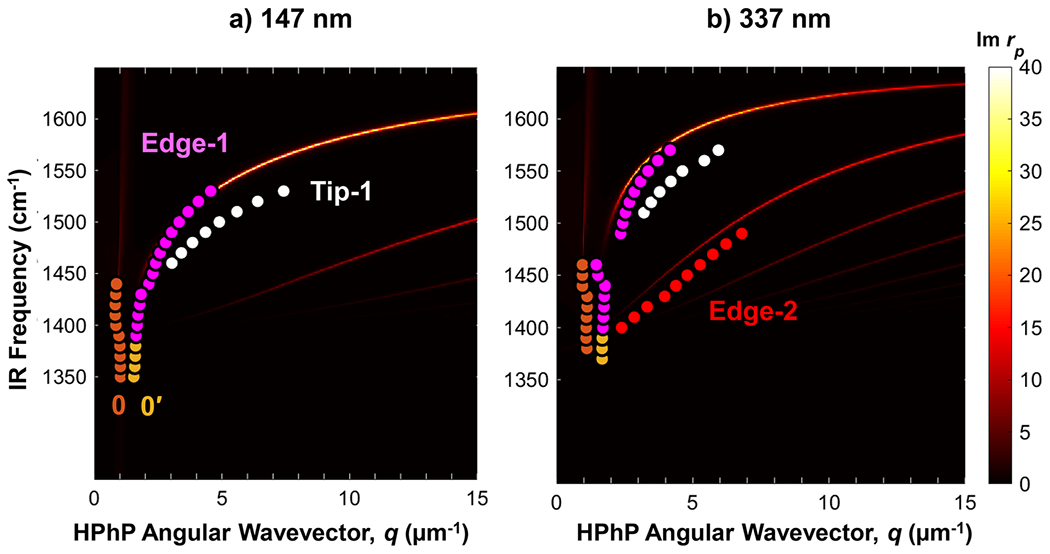
Comparison of the as-measured hyperbolic phonon polariton (HPhP) wavevectors, *q* (obtained from PTIR imaging analysis), with the calculated dispersion curves based on the dielectric function of the isotopically enriched B10≈99% hBN.^26^ Results are presented for flakes with thicknesses of (a) (a)≈147nm and (b) (b)≈337nm. The experimental data (solid circles) show dispersing branches with progressively increasing momenta and are labeled Edge-1, Tip-1, and Edge-2. Two low frequency modes are also detected and labeled $0,0^{\prime }$. The imaginary component of the computed reflectivity, Im $r_{p}$, is represented in the underlying color map and is used as a proxy for the theoretical HPhP dispersion.

A multi-mode damped harmonic oscillator model was fit to the reduced one-dimensional (1D) PTIR absorption profiles to extract the frequencies associated with each HPhP mode (see details in Fig. S3) as follows:

(1) $$\sum_{i}^{n}A_{i}e^{-\gamma_{i}x}\;cos\left(k_{i}x+\varphi_{i}\right),$$

where i indexes each of the n independent peaks evident in the Fourier power spectra [Fig. 1(d)], $A_{i}$ is a scaling coefficient, $\gamma_{i}$ is the damping coefficient, $k_{i}$ is the spatial peak center frequency $\left(\mu m^{-1}\right)$, and $\varphi_{i}$ is a phase shift that accounts for offsets in the relative position of the origin (determined topographically by the flake edge). The angular HPhP wavevector is defined as q=2π/Λ, where Λ represents the polariton wavelength and is extracted from either the spacing between edge-launched polaritons in the 1D profile or twice the spacing between the tip-launched polariton fringes. Evident by the roughly micrometer- and submicrometer-scale fringe spacings in real-space maps, the polaritons enable a strong confinement of light (up to ≈7-fold at $1560\;{cm}^{-1}$) with respect to the free-space wavelength $\left(\lambda_{IR}\right)$ (see Fig. S4). Subsequent Fourier analyses of the reduced line profiles yield the corresponding polariton wavevectors that, as a function of IR wavenumber, provide the material dispersion relation. In Fig. 2, we compare the experimental results with the analytically calculated reflectivity of ≈99.2% isotopically enriched hBN.^26^ The analytical dielectric function for B10≈99% was calculated using a harmonic oscillator model, which has been validated by reflectance measurements in similar isotopic purity material (see Table S1).^26^ Compared to naturally abundant hBN, both the previously measured Raman spectra and the fitted reflectance spectra show a significant spectral shift in the TO/LO phonon frequencies (as well as the damping factors) for the isotopically enriched samples.^26^ The comparison reveals multiple, thickness-dependent excitation branches, where a given free-space wavelength is compressed to shorter polariton wavelengths in each successive (higher-order) branch.^67^ The first branch, referred to as the fundamental mode (labeled as “Edge-1”), agrees well with the theoretical predictions for both flakes. For the thicker 337nm flake, we also observe the second-order polariton mode [Fig. 2(b); labeled “Edge-2”], consistent with previous observations.^26^ In addition to HPhPs, fringes with very small wavenumbers (labeled 0 and $0^{\prime }$ in Fig. 2) were observed in both flakes, which we attribute to waveguide modes and are discussed at the end of this manuscript.

Notably, for both flakes, an “extra branch” (denoted as “Tip-1”) is detected with momenta between the Edge-1 and Edge-2 branches. As mentioned previously, s-SNOM data on 2D hyperbolic flakes have revealed the coexistence of both tip- and edge-launched HPhPs, with the tip-launched modes having approximately double the measured frequency of the edge-launched variety.^26^ However, the ratio between Tip-1 and Edge-1 momenta ranges from about 1.26 (at $1460\;{cm}^{-1}$) to around 1.70 (at $1550\;{cm}^{-1}$) for the 147nm thick flake and between 1.27 (at $1510\;{cm}^{-1}$) and 1.42 (at $1570\;{cm}^{-1}$) for the 337nm thick flake [Fig. S5(a)]. These results suggest that the ratio is not constant (≈2) as expected. The effect of the light incident angles (α,β) on the s-SNOM measured fringe spacing has been reported for surface phonon polaritons in SiC^68,69^ and waveguide modes in MoS_2_^64^ and PtSe_2_;^65^ but it has not been typically considered for HPhPs in hBN since it was previously estimated to be insignificant.^61^ In addition, the effect of the flake edge orientation has been recently shown to affect the relative tip and edge HPhP launching efficiency in isotopically enriched (≈99.2%B11) hBN,^28^ but its effect on the fringe spacing has not been detailed. Here, the genuine (g) in-plane wavevectors of the edge-launched polaritons were calculated by accounting for the orientation angles as follows:^64^

(2) $$q_{\text{edge},\text{g}}=q_{\text{edge}}-q_{IR}[cos(\alpha )\times sin(\beta )],$$

where $q_{\text{edge}}$ is the wavevector extracted from the fringe spacing near the edge, $q_{IR}$ is the free-space wavevector of light, α [20°, Fig. 1(a)] is the illumination angle with respect to the sample plane, and β (90° for the PTIR measurements) is the angle between the direction of HPhP propagation and the sample edge [see Fig. 1(a)]. Note that the tip-launched HPhPs have been reported to be independent of the orientation of the illuminating field.^70^ After calculating the genuine edge-launched wavevectors with Eq. (2), we retrieve the expected ≈2:1 ratio for edge- and tip-launched polaritons [see Fig. S5(b)]. We believe that Eq. (2) has not been typically considered in the previous hBN HPhP literature because of the typically shorter scan windows employed (8−10μm) that are comparable with the in-plane component free-space wavelength. Here, we show that Eq. (2) is required to analyze the wide scan (40μm) data in our work and that such wide scan lengths are advantageous to accurately measure phonon-polariton characteristics, such as propagation length and lifetimes. Wide scan lengths have a higher resolution for Fourier space analysis $\left(0.05\;\mu m^{-1}\right.$ for a 40μm scan vs $0.25\;\mu m^{-1}$ for an 8μm scan) and can disentangle the effect of the incident free-space wavelength on the measured interference pattern. Consequently, large scans enable us to resolve the polariton peaks in Fourier space that are narrow, closely spaced, or low at frequency and to estimate more accurately the peak’s position and linewidth, which are directly related to the propagation length and lifetime (see Fig. S6 of the supplementary material). Due to the strong relative intensity of the edge-launched HPhPs in the samples measured here, Eq. (2) shall be used for comparing PTIR and s-SNOM data and to account for the different illumination conditions in each experimental setup. We also note that such differences may be more apparent in this paper due to the very long scans and HPhP propagation reported.

**FIG. 3.**
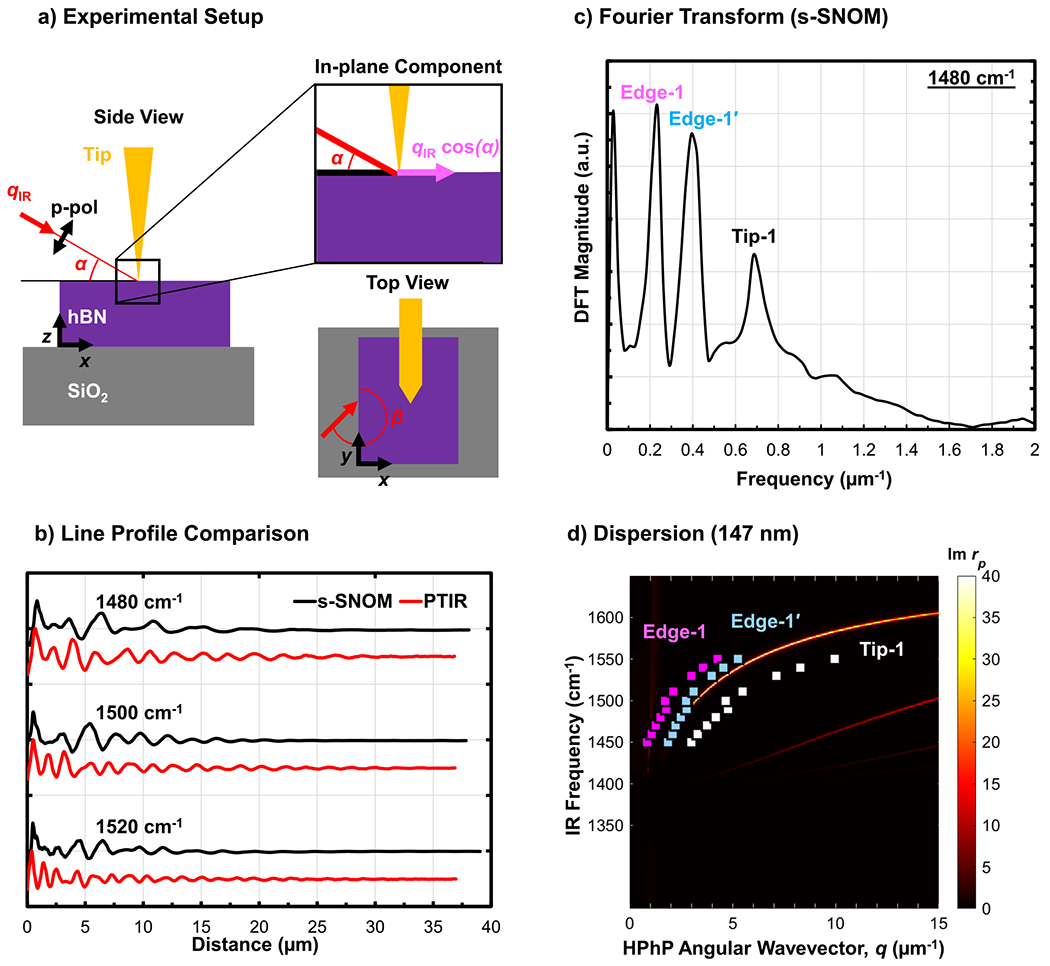
(a) Schematic illustrating sample illumination scheme in photothermal induced resonance (PTIR) experiments. Infrared (IR) light (red arrow with wavevector $q_{R}$) is incident upon the sample at an angle of α with respect to the plane of the sample and an angle β with respect to the flake edge. The magnified schematic outlines the in-plane and normal components of the p-polarization (p-pol) light. (b) Representative comparison of PTIR and s-SNOM column-wise averaged line scans at different incident IR frequencies. (c) Discrete Fourier transform (DFT) of the s-SNOM line profile revealing the HPhP spatial frequencies. (d) Comparison of extracted hyperbolic phonon polariton (HPhP) wavevectors, *q*, obtained from s-SNOM imaging to the calculated dispersion curves based on the [^10^B] 99.22% hBN dielectric function^26^ with 147 nm thickness.

To further corroborate the PTIR analysis, we measured the 147m thick hBN flake in a similar location to the PTIR measurements using s-SNOM, the prevalent method for polariton imaging. In the s-SNOM setup, the sample was illuminated with p-polarized light incident at $30^{\circ }$ with respect to the sample plane $\left(\alpha =30^{\circ }\right)$ and at $225^{\circ }$ with respect to the long axis of the AFM cantilever [see Fig. 3(a)]. The s-SNOM data were acquired by tapping a gold-coated AFM tip at 271−307kHz with an amplitude of 65−91nm and demodulating the second harmonic amplitude signal. The same data processing and Fourier analysis steps used for PTIR were conducted on the reduced s-SNOM line scans [e.g., Fig. 3(b)] to obtain the polariton wavevectors [Fig. 3(c)]. The theoretical dispersion relation determined from the computed material reflectivity method is then compared with the experimental data points in Fig. 3(d) with good quantitative agreement.

**FIG. 4.**
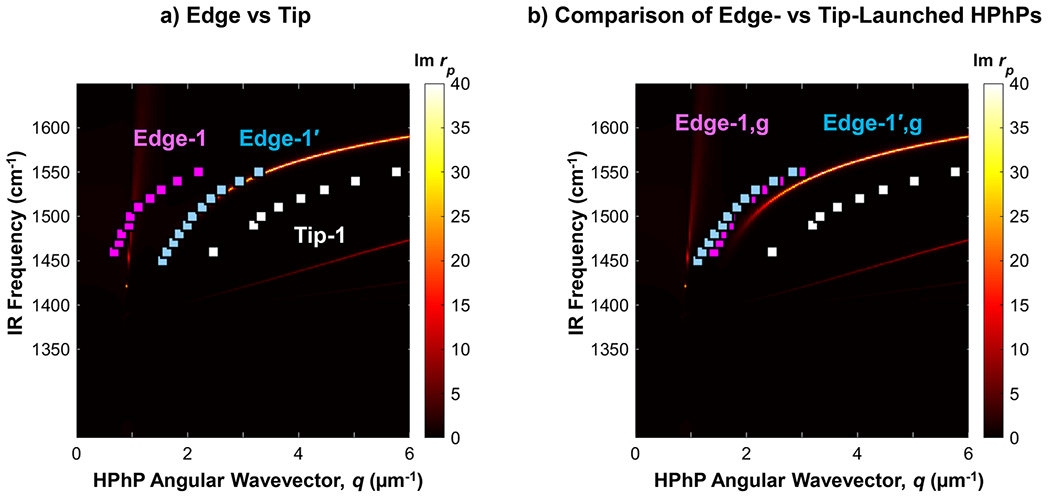
(a) Comparison of genuine HPhP wavevectors obtained from PTIR (circles) and s-SNOM (squares) imaging to the calculated dispersion curves based on the B1099.22% hBN dielectric function^26^ for a flake with 147 nm thickness. (b) The wavevectors of the genuine tip-launched HPhPs are halved to demonstrate the approximate 2:1 ratio measured by both techniques.

By applying Eq. (2) to the “Edge-1” branch shown in Fig. 3(d) $\left(\alpha =30^{\circ },\beta =230^{\circ }-238^{\circ }\right.$), we extract the expected 2:1 ratio (Fig. S9). Note that the s-SNOM line scans for this dataset were measured in two different sessions, which required manually repositioning the sample in the setup. The β angle was $230^{\circ }\pm 2^{\circ }$ for the first set of measurements $\left(1450-1490\;{cm}^{-1}\right)$ and $238^{\circ }\pm 2^{\circ }$ for the second set $\left(1500-1550\;{cm}^{-1}\right)$. Photographs of the sample in Fig. S10 depict the change in the angle β for the two sets of measurements. The effect of β on the as-measured polariton momenta (i.e., fringe spacing) is clearly visible as an offset in Fig. 3(d) but is corrected by calculating the genuine momenta with Eq. (2) [Fig. 4(a)], corroborating our analysis. Both the genuine “Edge-1” and “Tip-1” branches from the s-SNOM experiments agree well with the branches determined with PTIR on this flake.

One peculiar difference between the s-SNOM and PTIR datasets is the unexpected detection of an additional branch near the first edge-launched branch [denoted as “Edge-1′” in Fig. 3(d)] in the s-SNOM data. This feature is detected when considering the whole 40μm wide window, and it is not detected when truncating the data to a 10μm scan due to the insufficient resolution in Fourier space [see Fig. S6(b)]. Notably, calculating the $dq_{\text{Tip}-1}/dq_{\text{Edge}}$ gradient for each branch reveals gradients of ≈2.0 for both Edge-1 and Edge-1′ modes [Fig. S9(a)], suggesting that both branches should be of the edge-type and of the same order. We note that the Edge-1′ mode matches the genuine Edge-1 dispersion according to the following equation:

(3) $$q_{\text{edge},g}=q_{{\text{edge}1}^{\prime }}+\frac{q_{IR}}{2}[cos(\alpha )\times sin(\beta )].$$

Applying Eq. (3) to the Edge-1′ branch, the resulting genuine edge polaritons align extremely well with the Edge-1,g data for both s-SNOM and PTIR (Fig. 4). However, we have not identified which physical mechanism underlies such a modified interference pattern. As a control, we use s-SNOM to measure a different hBN flake $\left(\approx 280\;nm\right.$ thick $\left.\alpha =30^{\circ },\beta =247^{\circ }\right)$ and obtained consistent results for all three branches (see Figs. S8 and S9).

**FIG. 5.**
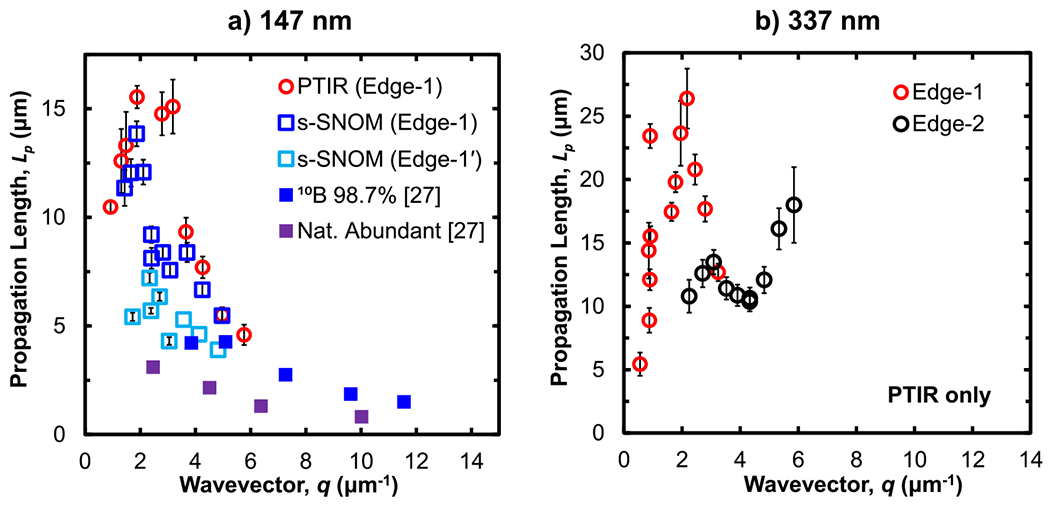
(a) Propagation lengths, $L_{p}$, for Edge-1 polaritons in an ≈147nm thick B10≈99% hBN flake via PTIR (open circles) and s-SNOM (open squares). Propagation lengths are plotted as a function of the genuine in-plane wavevector, *q*, and compared to the previous s-SNOM measurements^26^ for an ≈120nm thick B1098.7% hBN flake (solid blue) and natural abundant hBN (solid purple). (b) Propagation lengths for Edge-1 (red circles) and Edge-2 (black circles) branches as identified in Fig. 2(b) for an ≈337nm thick B10≈99% hBN flake, as a function of *q*. The error bars represent the propagated uncertainty in the values of the fitted parameters (95% confidence intervals) used to calculate the propagation length.

Next, we extract useful parameters from the data to evaluate and benchmark the characteristics of the measured HPhPs. For each mode, we calculate the propagation length $\left(L_{p}=1/\gamma_{n}\right)$, where $\gamma_{n}$ is the harmonic damping coefficient, and compare the obtained values with prior results on naturally abundant and isotopically enriched hBN (see Fig. 5). While previous s-SNOM measurements^26^ revealed $L_{p}<4\;\mu m$, for a 120nm thick, $h^{10}BN$ (98.7%) flake, here, we obtain propagation lengths up to ≈14μm (s-SNOM) and up to ≈15μm (PTIR) for an ≈147nm thick flake of the similar nominal isotopic composition [see Fig. 3(b) for a direct comparison of PTIR and s-SNOM absorption profiles]. Such long propagation lengths can be intuitively confirmed by visually inspecting the polaritonic fringe patterns in Fig. 1, which demonstrates the benefit of isotopic enrichment in hBN of ≈5× with respect to the measurements on naturally abundant materials. Record propagation lengths exceeding ≈25μm (at room temperature) were evident in PTIR measurements on an ≈377nm thick flake [Fig. 5(b)]. In agreement with previous results,^17^ the greater optical confinement (larger q) of the second-order mode detected in the thicker (≈337nm) flake generally results in shorter but still remarkably long propagation lengths of about $10\;\mu m<L_{P2}<17\;\mu m$ [Fig. 5(b)].

To benchmark HPhP performance for nanophotonic applications more quantitatively, next we focus on the fundamental polariton branch and compute the polariton group velocity $v_{g}=\partial \omega /\partial q$ from the slope of the dispersion curve in Fig. 2 (see also Fig. S7) and its lifetime (τ) defined as follows:

(4) $$\tau =\frac{L_{p}}{v_{g}}.$$

Another useful benchmark that enables comparing polaritons from different materials is the propagating figure of merit (the Q of the polariton in Fourier space) defined as

(5) $$Q=\frac{Re(q)}{Im(q)},$$

where Re(q) is the in-plane wavevector and Im(q) is proportional to the fitted half-width at half-maximum of the spectral linewidth extracted from the power spectra in Fourier space.

**FIG. 6.**
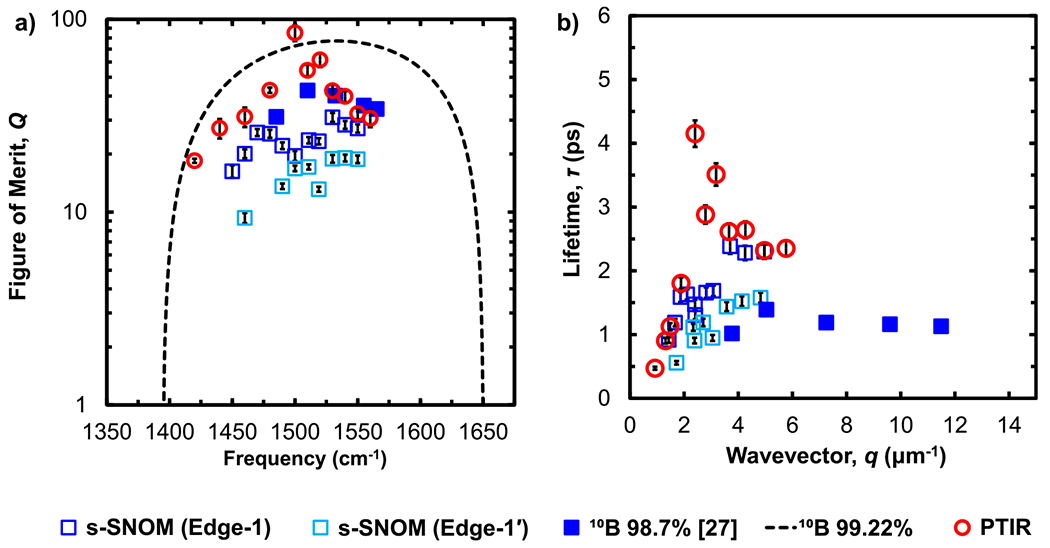
(a) Figure of merit, Q, representing the quality of the polaritons and (b) lifetimes, τ, for B10≈99% isotopically enriched hBN flakes with a thickness of ≈147nm estimated via PTIR (open red circles) and s-SNOM (open blues squares) and compared to previous s-SNOM measurements^26^ for an ≈120nm thick flake of similar composition (B1098.7% hBN, blue solid squares).^26^ The error bars represent the uncertainty propagation of the fitting parameters (95% confidence intervals) used to calculate the Q and lifetime.

The resulting Q’s and HPhP lifetimes are plotted in Fig. 6 and compared with previously reported values measured using s-SNOM on flakes of similar composition and thicknesses.^26^ The Q and the polariton lifetimes (up to ≈4ps) obtained for these large, isotopically enriched flakes approach the values predicted by theory^24^ for B10 hBN and exceed the theoretical limits for naturally abundant hBN (τ≈1.5ps^24^). The long lifetimes measured here support the hypothesis that the previously reported values for these samples^26^ were constrained by the lateral hBN flake size, which we overcome here using flakes with lateral dimensions that significantly exceed the HPhP propagation lengths and using 40μm wide scan lengths.

In addition to HPhPs, the PTIR images reveal another set of branches in the hBN dispersion (labeled as “0” and “0′” in Fig. 2) that we believe have not been reported previously. These branches are associated with small wavevectors $\left(\approx 1\;\mu m^{-1}\right)$ that do not disperse strongly as a function of the incident IR frequency and match well with the expected light line calculated by the reflectivity model (Fig. 2). We attribute these features to waveguide-like modes (sometimes referred to as air modes)^64^ in hBN that display low optical compression and remarkably long propagation lengths (Fig. S11). Interestingly, these waveguide-like modes are also detected below the TO phonon frequency $\left(\approx 1390\;{cm}^{-1}\right)$ and thus outside the Reststrahlen band, down to around $1350\;{cm}^{-1}$ (see Fig. 2). Note that while two modes can be distinguished clearly outside the Reststrahlen band, the higher momentum mode of the two closely overlaps in momentum space with the Edge-1 mode within the Reststrahlen band, making their distinction challenging. However, we note that $L_{p}$ and Q as a function of frequency for the Edge-1 and $0^{\prime }$ modes show a sharp discontinuity in correspondence with the hBN phonon mode at $1390\;{cm}^{-1}$, which we leveraged to classify those two modes.

## CONCLUSIONS

The analysis of hyperbolic polaritons in large, isotopically enriched hBN flakes (up to 250μm), free of macroscopic defects, with wide scan (40μm) PTIR and s-SNOM maps enables precise $\left(0.05\;\mu m^{-1}\right.$ resolution) quantification of the key characteristics, including dispersion relation, propagation length, group velocity, lifetime, and figure of merit. Our results on ≈99%B10 hBN show an eightfold improvement of polariton propagation lengths (up to ≈25μm) and a fourfold improvement in lifetimes (up to ≈4.2ps) with respect to naturally abundant samples and a record-breaking propagating figure of merit (Q≈90) matching earlier theoretical predictions. These results confirm the hypothesis that previous s-SNOM measurements of the polariton lifetimes and propagation lengths on these samples were, in part, artificially restricted by the flake width. With lifetimes close to theoretically predicted values, our analysis confirms the dominant role of the isotopic disorder over other decay channels (anharmonic decay and background impurities) in determining the lifetimes of optic phonons in hBN. Therefore, further improvements shall be expected only for isotopically purest samples with even lower background impurities. The analysis and methods presented here can be generally applied to other van der Waals crystals and their heterostructures to benchmark polariton performance for a variety of nanophotonic applications. Particularly, we show that the period of the measured polariton fringes in near-field maps is affected by the light incident angles with respect to the sample plane (α) and with respect to the flake edges (β). These angles shall be accounted for when comparing measurements obtained with different measurement schemes or on flakes with edges illuminated from different angles. Even more importantly, we show that to extract accurate lifetimes and propagation lengths, large scan lengths (≫ propagation length and in-plane incident wavelength) on large flakes that are free of other defects or asperities (which also can launch the polaritons) are necessary. Overall, the extremely long propagation lengths of the polaritons studied here and the material growth-based strategy will enable progress in the development of nanophotonic materials and devices toward the use in hyperlensing and ultra-thin optics applications.

## Supplementary Material

Supp1

## Data Availability

The data that support the findings of this study are available within the article and its supplementary material and from the corresponding author upon reasonable request.
