# Supplementary material for: Experimental confirmation of long hyperbolic polariton lifetimes in monoisotopic (10B) hexagonal boron nitride at room temperature
Source: APL Mater. Author manuscript; Available in PMC 2023 Sep 15. (PMC10502608; doi:10.1063/5.0061941)
Supplement: Supp1 [file NIHMS1918299-supplement-Supp1.docx]

Supplemental Material:

**Experimental Confirmation of Long Hyperbolic Polariton Lifetimes in Monoisotopic (10B) Hexagonal Boron Nitride at Room Temperature**

Georges Pavlidis,^1^ Jeffrey J. Schwartz,^1,2^ Joseph Matson,^3^ Thomas Folland,^3^ Song Liu,^4^ James H. Edgar,^4^ Joshua D. Caldwell,^3^ Andrea Centrone^*,1^

*^1^*Physical Measurement Laboratory, National Institute of Standards and Technology, Gaithersburg, Maryland 20899, United States

*^2^*Institute for Research in Electronics and Applied Physics, University of Maryland, College Park, Maryland 20742, United States

*^3^*Mechanical Engineering, Vanderbilt University, Nashville, TN, 37235, USA

*^4^*Tim Taylor Chemical Engineering, Kansas State University, Manhattan, KS, 66506, USA

Contact Information:

*E-mail: [andrea.centrone@nist.gov](mailto:andrea.centrone@nist.gov)

**Photothermal Induced Resonance (PTIR)**

The PTIR measurements performed in this study were completed using a tapping-mode heterodyne measurement paradigm. A gold-coated Si probe was used with a nominal spring constant of 0.07–0.4 N/m. For image recording, 40 µm line scans were measured at rates of 0.01–0.02 Hz with a 1000-pixel resolution. The tip and hBN surface were illuminated using a quantum cascade laser array with a tunable pulse repetition rate. The laser spot was centered around the probe tip and approximated to have a 50 µm diameter. Absorption maps were measured by scanning the probe while illuminating the sample at a constant wavelength.

**Effect of Polarization and Orientation of Edge**

**
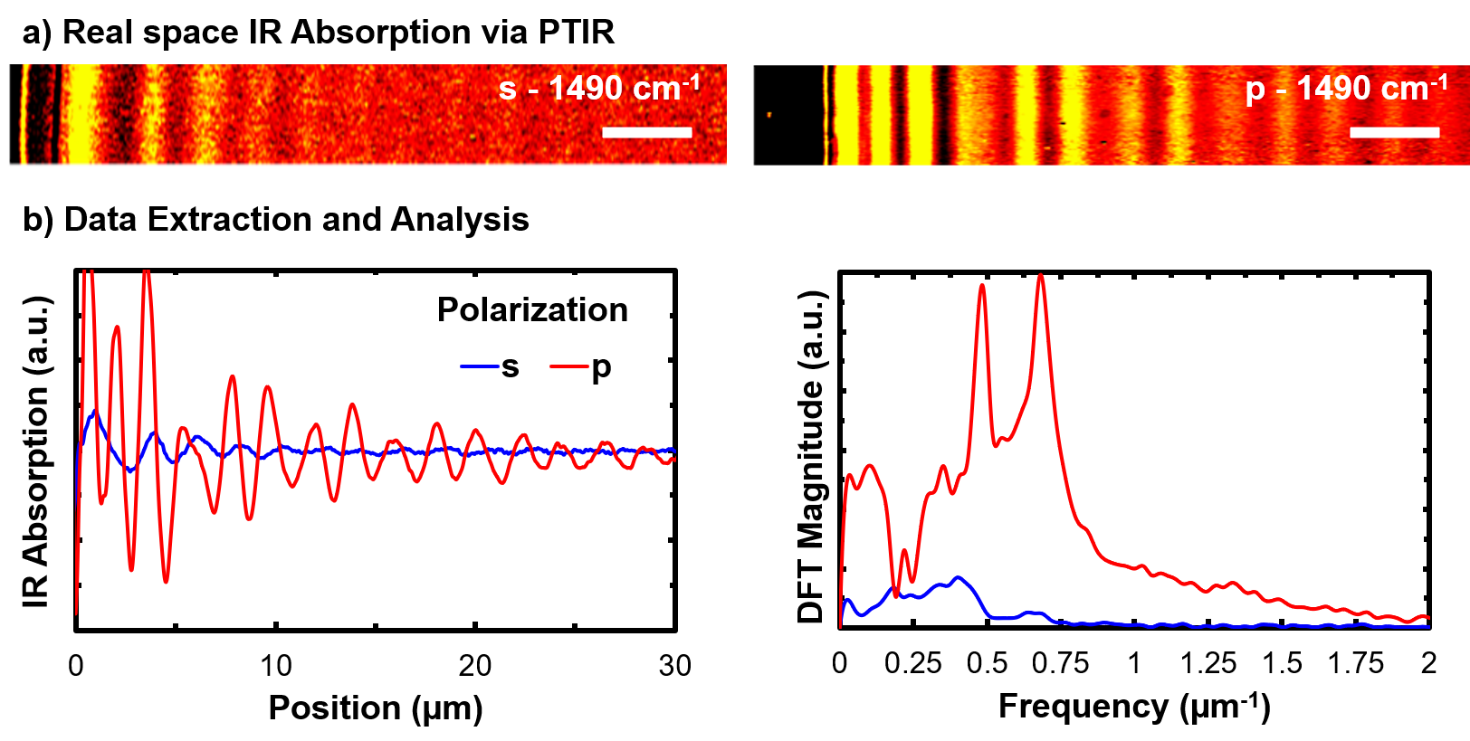
**

FIG. S1. a) Photothermal induced resonance (PTIR) absorption images of hyperbolic phonon polaritons (HPhPs) in a ≈ 337 nm thick isotopically enriched (^10^B ≈ 99 %) hBN flake obtained by illuminating the sample at 1480 cm^-1^ with s-polarization (left) or p-polarization (right). The scale bars represent 5 μm. For each image, line scans were averaged and plotted as a function of the distance from the flake edge. b) Column-wise averaged absorption at 1480 cm^‑1^ (left) and its discrete Fourier transform (DFT) revealing the detected HPhP frequencies (right).

**
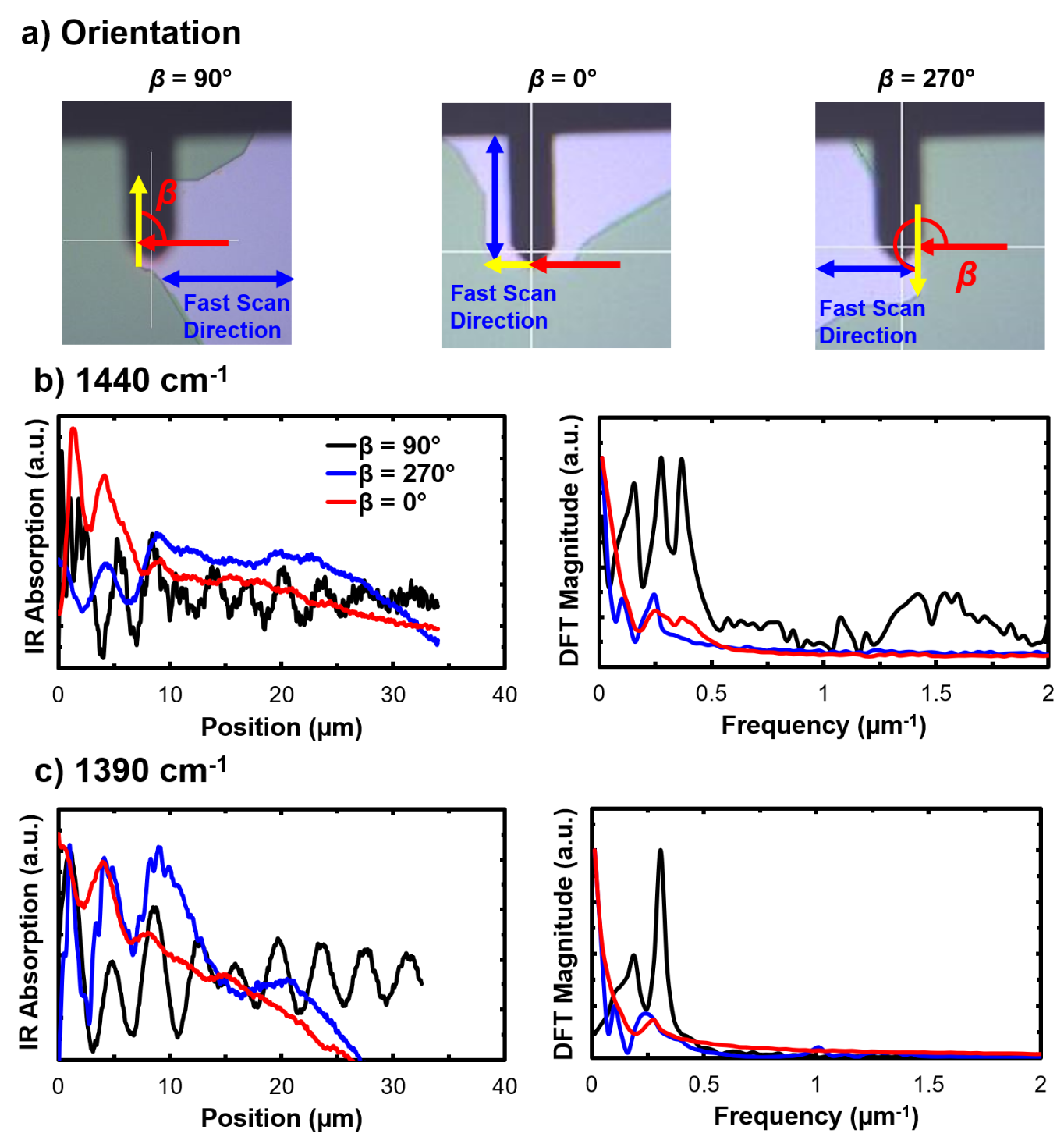
**

FIG. S2. a) Photographs depicting the in-plane orientation of a ≈ 147 nm thick isotopically enriched (^10^B ≈ 99 %) hBN flake for edge orientation angles of β = 90°, 0° and 270°, respectively. The blue arrows represent the fast-scan direction of the probe, the red arrows are the direction the incident IR light, and the yellow arrows indicate the orientation of the flake. Examples of averaged PTIR absorption profiles (left) and discrete Fourier transform (DFT, right) for incident laser frequencies of b) 1440 cm^‑1^ and c) 1390 cm^‑1^

**Data Fitting and Processing**

Topography images were first flattened and then averaged in the vertical direction (parallel to the crystal edge). Next, we obtained an accurate estimate of the flake edge position by applying a Canny edge detection algorithm to the averaged topographic line profile. From this step onwards, all further analysis was conducted on the data inside the flake edge as plotted in Fig. S3a.

**
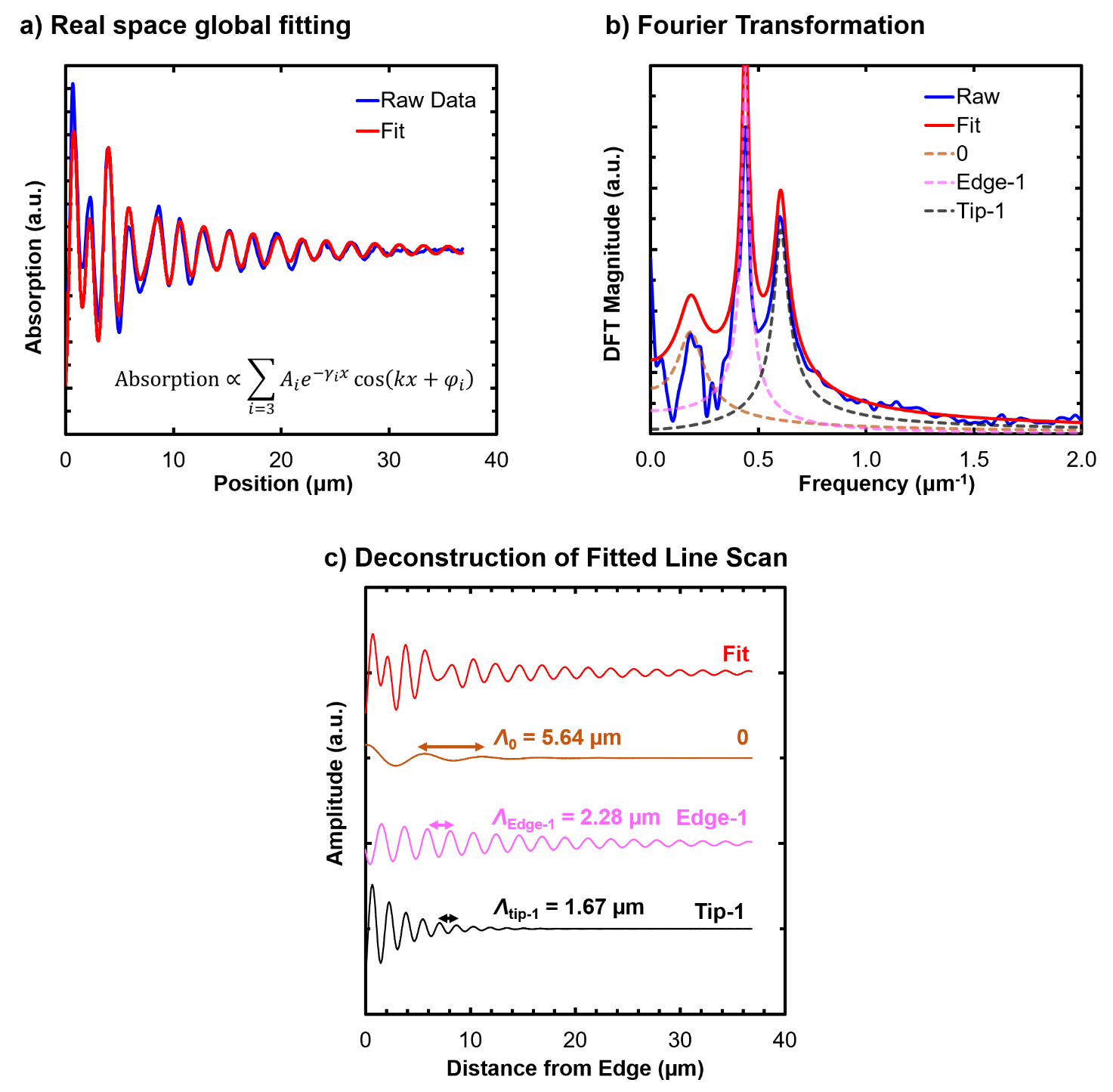
**

FIG. S3. a) Averaged PTIR absorption line profiles at each wavelength were fit in the spatial domain to a multi-component damped harmonic oscillator defined by the inset equation. For this scan, three modes were fitted and are clearly observed in the b) frequency domain. Individual components of the global fit are plotted to portray more clearly the presence of different HPhP modes present. c) The global fit and the individual components are also plotted in real space to portray clearly the fringe spacing, Λ.

Next, the line scans were converted to the frequency domain, where HPhPs peaks were visually identified (Fig. S3b). Individual peak fits were performed to obtain initial estimates of fitting parameters for each peak. A global fit to a multi-component harmonic damped oscillator was performed and plotted in Fig. S3a. The equation (Eq. 2 of the main text) for the damped harmonic oscillator is shown inset in Fig. S3a. The fitted data were then transformed back into the frequency domain to visually inspect fit with respect to the peak position and peak width. Both components are critical for assessing the propagation characteristics associated with the HPhPs, which include the in-plane wavevector and the propagation length.


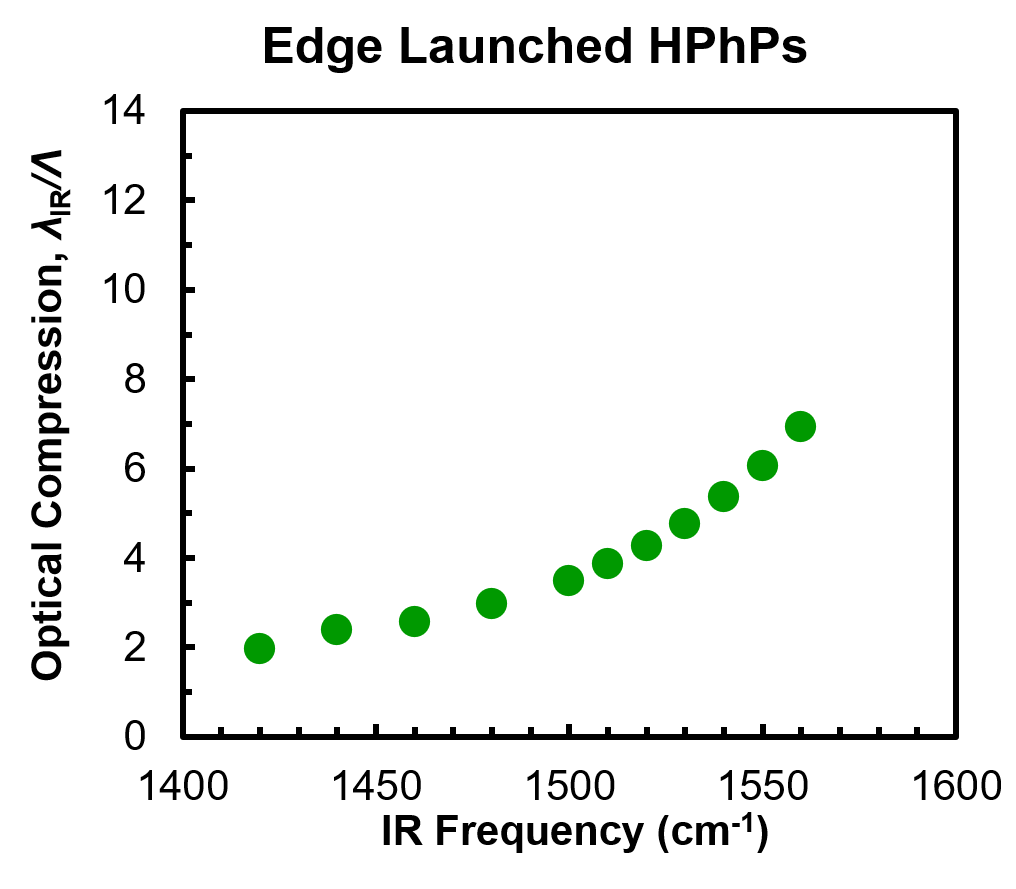


FIG. S4. Calculated light compression of free space wavelength (λ_IR_) relative to the HPhPs wavelength (Λ) in an isotopically enriched (^10^B ≈ 99 %) hBN flake (≈ 147 nm thick). The HPhP wavelengths were obtained from PTIR data for the Edge-1 branch. The error bars (smaller than the markers) represent the propagated uncertainty of the fitting parameters (95% confidence intervals).

**Parameters for the Infrared Dielectric Function**

TABLE S1. High frequency permittivity (ε_∞_), transverse optical (TO) and longitudinal optical (LO) phonon frequencies (ω_TO_, ω_LO_), and the phonon damping factor (Γ) for ^10^B ≈ 99 % hBN used in this work for the analytical calculations.^27^

| ^10^B 99.2 % hBN | *ε*_∞_ | *ω*_TO_ (cm^-1^) | *ω*_LO_ (cm^-1^) | *Γ* (cm^-1^) |
| --- | --- | --- | --- | --- |
| Ordinary | 5.1 | 1394.7 | 1650.2 | 1.8 |
| Extraordinary | 2.5 | 785.1 | 845.1 | 1 |

**Tip vs. Edge Analysis**

Fig. 2 of the main text shows two HPhP branches near the first theoretical branch. The branch labelled as Edge-1 is shown to agree with the theoretical curve while the branch labelled as Tip-1 appears as an offset to this branch. The wavevector ratios for these two branches were calculated and plotted in Fig. S5a.

**
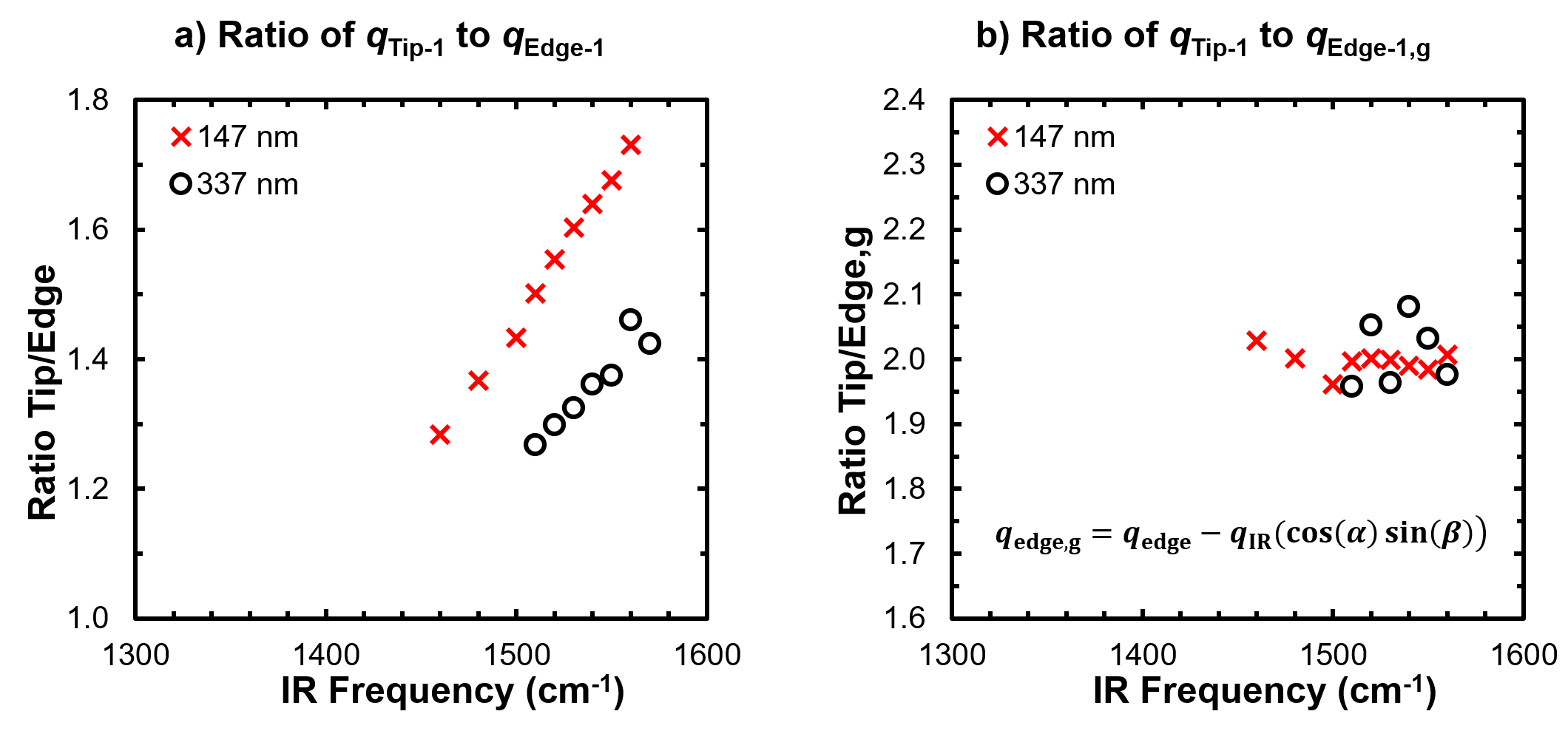
**

FIG. S5. As measured, wavevector ratio of the Tip-1 and Edge-1 mode as function of incident wavelength. The ratio increases with increasing wavenumber. b) Genuine, wavevector ratio of the Tip-1 and Edge-1 modes after correcting for the incident angle and flake edge orientation showing the expected 2:1 ratio.

**Effect of Scan Range on Discrete Fourier Transform (DFT) Analysis**

**
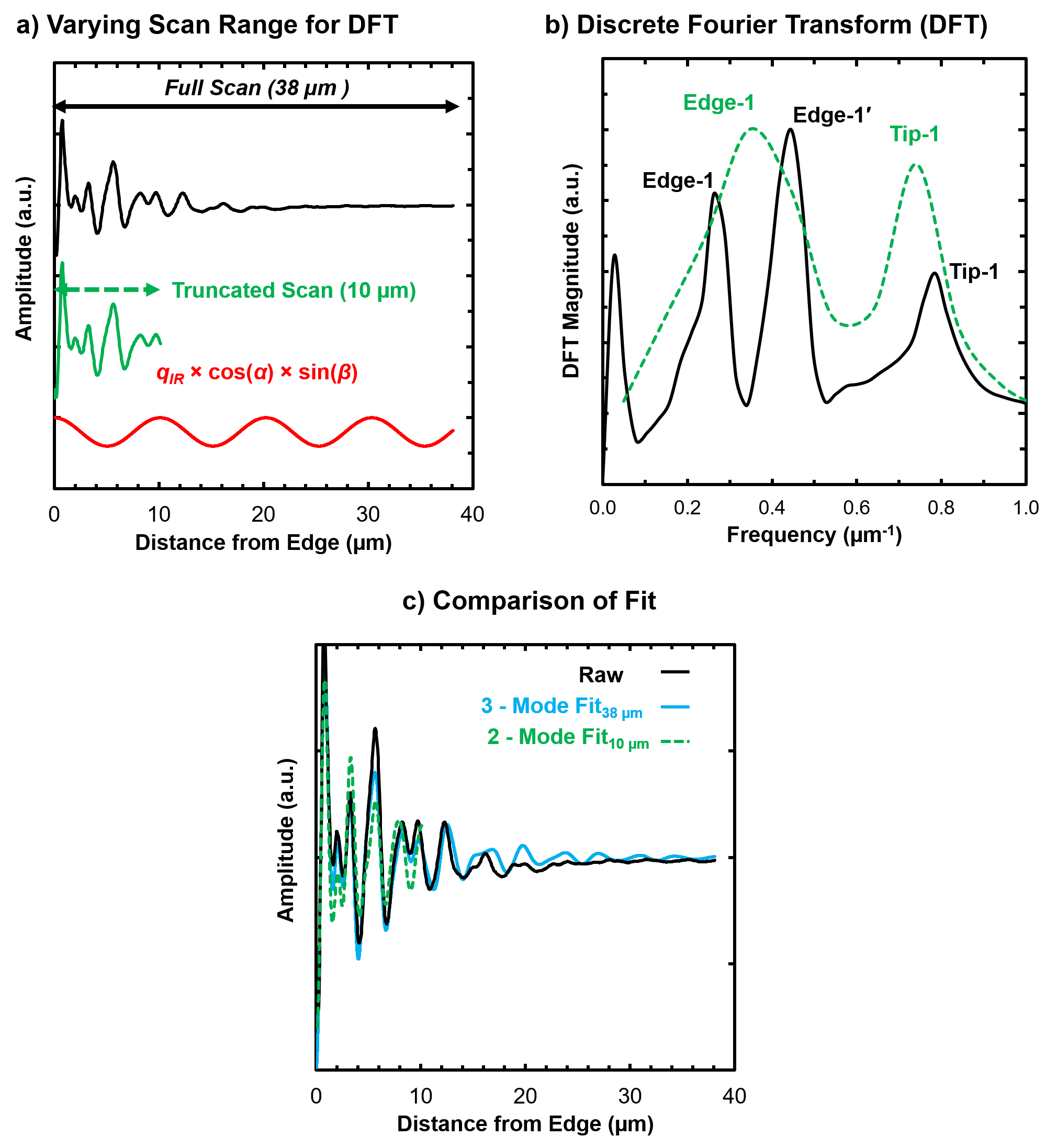
**

FIG. S6 a) Column averaged s-SNOM line profile at 1490 cm^-1^ for the full measurement scan (≈ 38 µm, black) and 10 µm truncated scan (green), which is comparable to the in-plane free space wavevector (wave*v*ector of the red trace) b) Comparison of the Discrete Fourier transform (DFT) power spectra for the full (≈ 38 µm, black) scan revealing 3 main peaks and truncated (10 µm, green) scan revealing 2 peaks. c) The global fittings of a *multi*-mode damped harmonic oscillator (Eq. 1) are overlaid and compared to the line profile (black) for the full scan range (*3-Mode* Fit_38µm_, blue solid line) and *t*he truncated scan range (*2-Mode* Fit_10µm_, green dashed line).

**Group Velocity**

The HPhP dispersion was plotted (Fig. 2) after calculating all the measured in-plane wavevectors. The group velocities (slope of the dispersion curves) were then estimated (Fig. S7) by using numerical differentiation and used to estimate the HPhPs lifetimes.

**
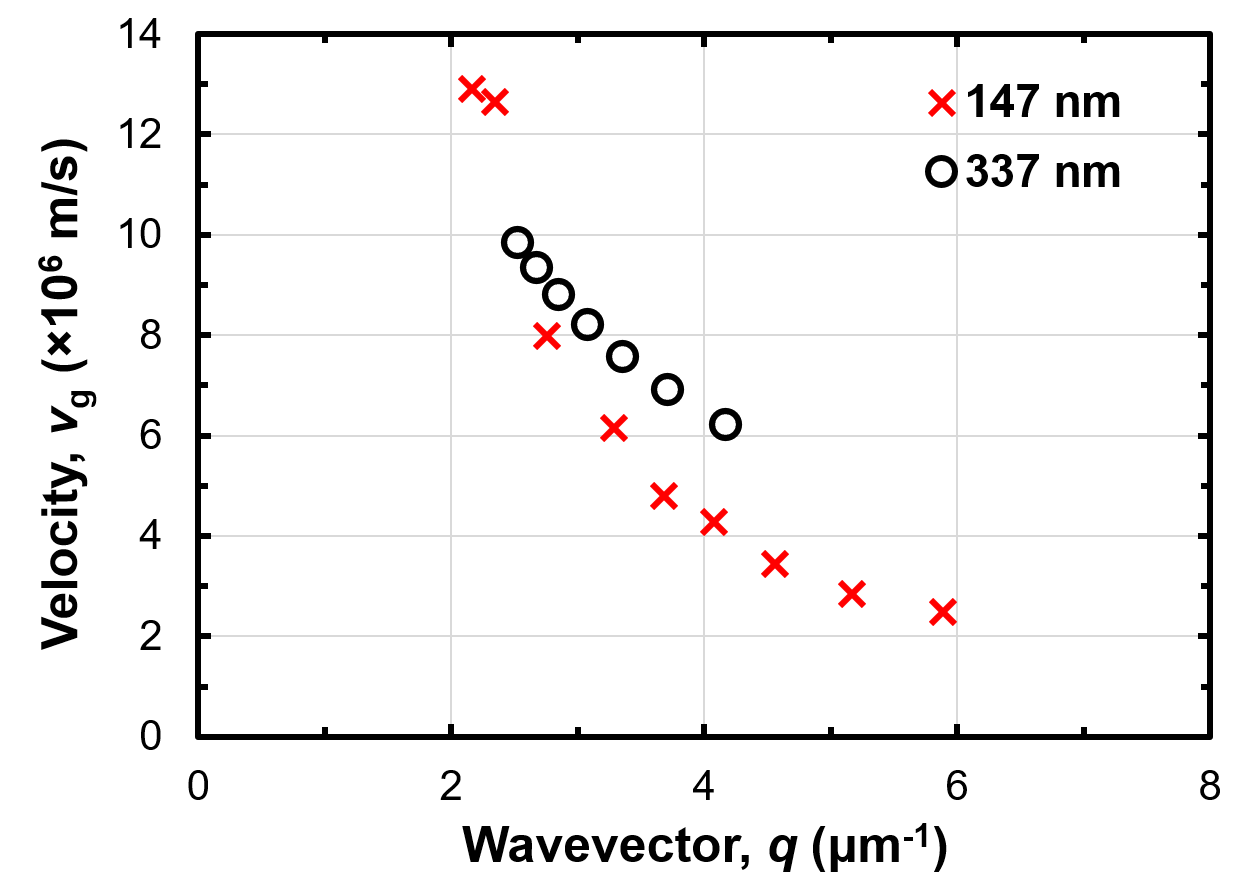
**

FIG. S7. Group velocities used to estimate lifetimes of edge-launched HPhP detected in a ≈ 147 nm and ≈ 337 nm thick isotopically enriched (^10^B ≈ 99 %) flakes. The values were calculated by numerical differentiation of the dispersion relationship obtained from PTIR data in Fig. 2.

**s‑SNOM Analysis**

The s‑SNOM measurements performed in this study were completed using a commercial system. The images are taken using a pseudo-heterodyne detection scheme. The tip and hBN surface were illuminated using a quantum cascade laser. The gold-coated AFM tip is operated in tapping mode, with a tapping frequency around 270 kHz. The second harmonic amplitude is used for analysis.

**
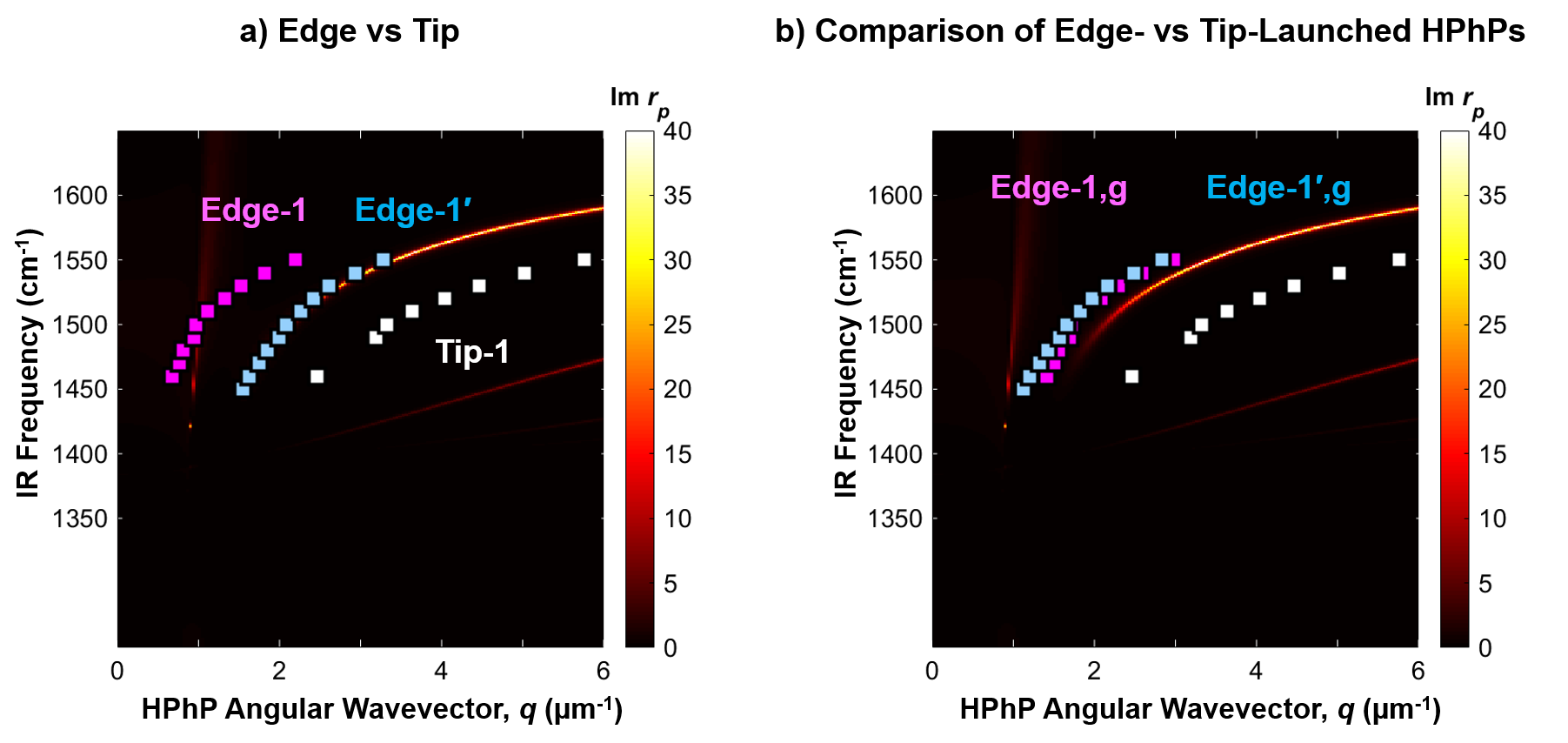
**

FIG. S8 a) Experimentally determined wavevectors (Edge-1, Edge-1′, Tip-1) as measured by s-SNOM from a ^10^B ≈ 99 % hBN ≈ 280 nm thick flake. b) genuine HPhPs wavevectors (Edge-1,g and Edge-1′,g) calculated using Eq. 2. Both sets of data are compared to the calculated dispersion curves based on the [^10^B] 99.22 % hBN dielectric function^27^ with 280 nm thickness.

**
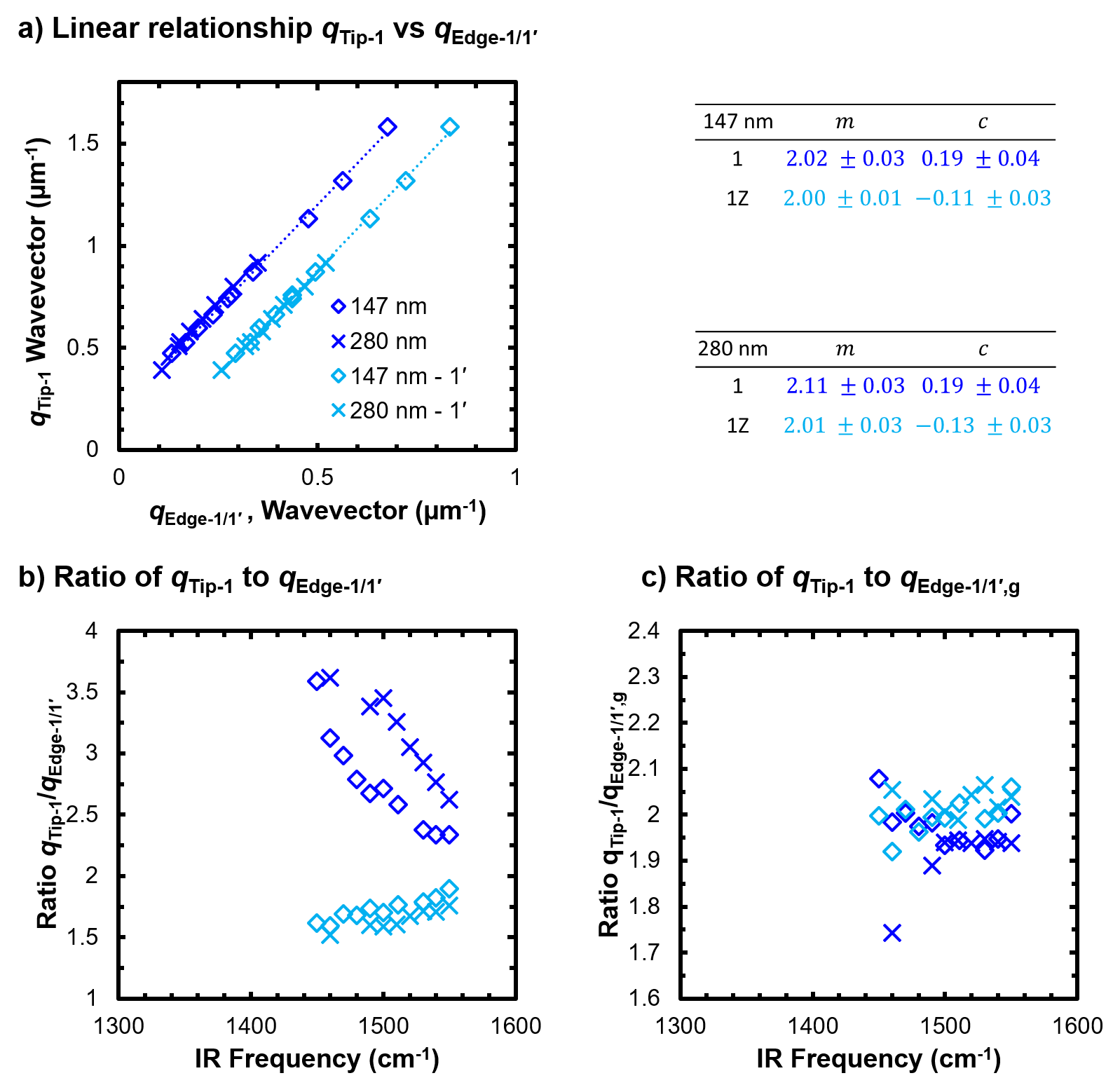
**

FIG. S9. a) Plot of the s‑SNOM detected tip launched wavevector (Tip-1) against the two-edge launched wavevectors (Edge-1 P/S) that show a linear relationship for two isotopically enriched (^10^B ≈ 99 %) flakes. The flake thicknesses are ≈ 147 (hollow diamonds) and ≈ 280 nm (crosses) b) The ratio of the as-measured tip launched vs edge launched wavevectors as function of incident wavelength. The ratio increases with increasing wavenumber. c) The ratio of the genuine (i.e., corrected using Eq. 2) tip launched vs edge launched wavevectors as function of incident wavelength.

**
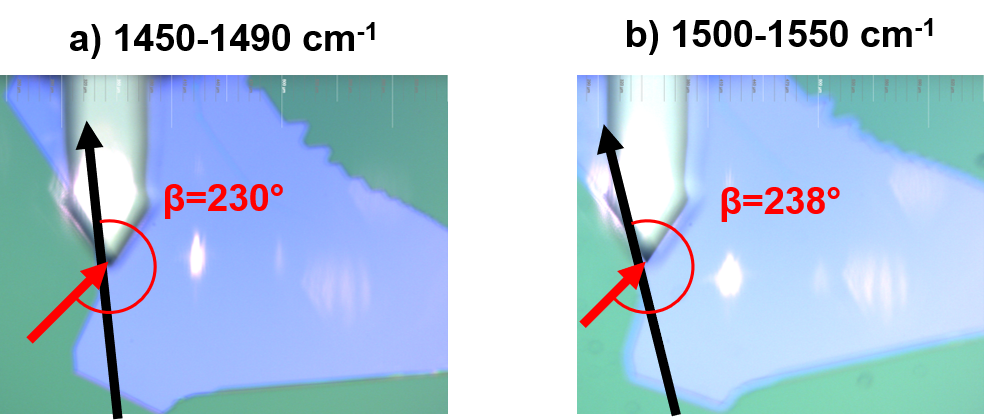
**

FIG. S10. Microscope optical images highlighting the different *β* angle during two subsequent s-SNOM measurement sets for a ≈ 147 nm thick isotopically enriched (^10^B ≈ 99 %) flake. *β* was constant for measurements in the spectral ranges between a) 1450 cm^-1^ – 1490 cm^‑1^ and b) 1500  cm^-1^ – 1550 cm^-1^. The sample was removed from the setup and reinstalled in a similar position between the two sets of measurements.

**Low Frequency Modes Detected in PTIR**

**
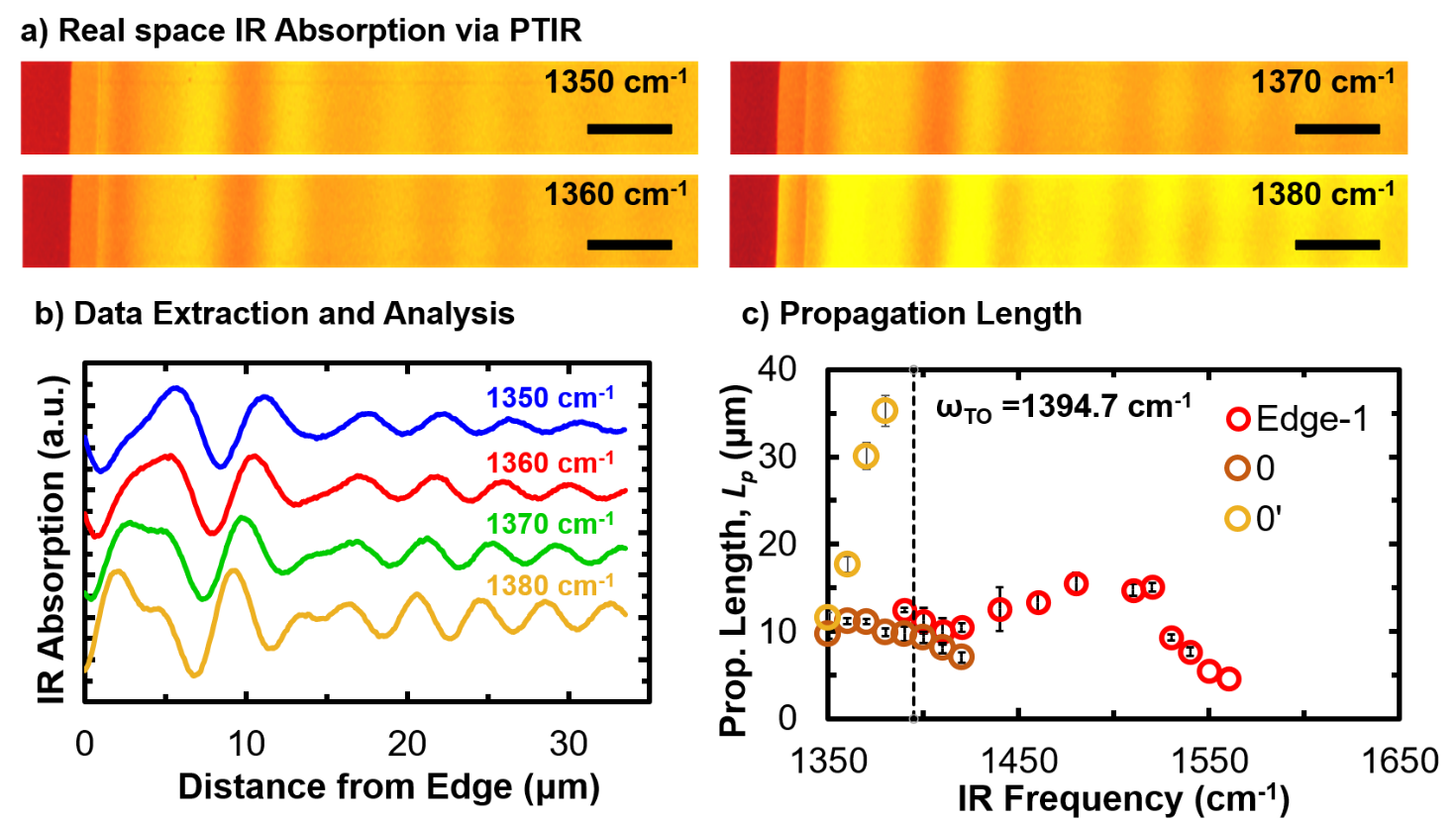
**

FIG. S11. a) Real space PTIR images of HPhPs in a flake of isotopically enriched (^10^B 99 %) hBN with thickness ≈ 147 nm obtained by illuminating the sample with wavelengths below the Reststrahlen band (<1390 cm^-1^). The scale bars represent 5 μm. b) Column-wise averaged absorption line scans corresponding to the images in panel-a. c) Calculation of propagation lengths, *L*_p_, with the corresponding IR frequency.
